# Hypertonic glucose inhibits growth and attenuates virulence factors of multidrug-resistant *Pseudomonas aeruginosa*

**DOI:** 10.1186/s12866-020-01889-2

**Published:** 2020-07-09

**Authors:** Tao Chen, Ye Xu, Wenya Xu, Wenli Liao, Chunquan Xu, Xiucai Zhang, Jianming Cao, Tieli Zhou

**Affiliations:** 1 Department of Clinical Laboratory, the First Affiliated Hospital of Wenzhou Medical University, Wenzhou, Zhejiang Province China; 2 School of Laboratory Medicine and Life Sciences, Wenzhou Medical University, Wenzhou, Zhejiang Province China

**Keywords:** Hypertonic glucose, *Pseudomonas aeruginosa*, Quorum sensing, Virulence factors, Biofilm, Motility

## Abstract

**Background:**

*Pseudomonas aeruginosa* is the most common Gram-negative pathogen responsible for chronic wound infections, such as diabetic foot infections, and further exacerbates the treatment options and cost of such conditions. Hypertonic glucose, a commonly used prolotherapy solution, can accelerate the proliferation of granulation tissue and improve microcirculation in wounds. However, the action of hypertonic glucose on bacterial pathogens that infect wounds is unclear*.* In this study, we investigated the inhibitory effects of hypertonic glucose on multidrug-resistant *P. aeruginosa* strains isolated from diabetic foot infections. Hypertonic glucose represents a novel approach to control chronic wound infections caused by *P. aeruginosa*.

**Results:**

Four multidrug-resistant *P. aeruginosa* clinical strains isolated from diabetic foot ulcers from a tertiary hospital in China and the reference *P. aeruginosa* PAO1 strain were studied. Hypertonic glucose significantly inhibited the growth, biofilm formation, and swimming motility of *P. aeruginosa* clinical strains and PAO1. Furthermore, hypertonic glucose significantly reduced the production of pyocyanin and elastase virulence factors in *P. aeruginosa*. The expression of major quorum sensing genes (*lasI*, *lasR*, *rhlI*, and *rhlR*) in *P. aeruginosa* were all downregulated in response to hypertonic glucose treatment. In a *Galleria mellonella* larvae infection model, the administration of hypertonic glucose was shown to increase the survival rates of larvae infected by *P. aeruginosa* strains (3/5).

**Conclusions:**

Hypertonic glucose inhibited the growth, biofilm formation, and swimming motility of *P. aeruginosa*, as well as reduced the production of virulence factors and quorum sensing gene expression*.* Further studies that investigate hypertonic glucose therapy should be considered in treating chronic wound infections.

## Background

*Pseudomonas aeruginosa* is an important human opportunistic pathogen that can cause life-threatening infections, particularly in immunocompromised patients. It is also the most common Gram-negative pathogen responsible for infections of chronic wounds, such as venous leg ulcers, pressure ulcers, and diabetic foot ulcers [1–3]. Extensive use of antibiotics has created an evolutionary pressure on *P. aeruginosa*, which has led to high levels of multiple drug resistance (MDR). Therefore, rational strategies that minimize the probability for the emergence of antibiotic resistance are needed to combat pathogenic *P. aeruginosa.*

Hypertonic glucose, which is a commonly used prolotherapy solution [4], can accelerate the proliferation of granulation tissue and improve microcirculation within a wound, which reduces the patient’s healing time and the requirement for further medical care [5]. The combined application of hypertonic glucose and negative pressure wound therapy was found to be safe and effective in preparing *P. aeruginosa*-infected wounds for grafting [6]. However, despite several reports indicating that hypertonic glucose is an effective therapy for chronic wound injuries, less is known regarding the actions of hypertonic glucose in the treatment of bacterial wound infections.

The pathogenicity of *P. aeruginosa* is influenced by processes such as biofilm formation, motility, and virulence factors such as pyocyanin and elastase [7–9]. These factors are regulated by quorum sensing (QS), which is a cell-cell communication system that monitors bacterial population density and coordinates physiological processes accordingly [10, 11]. *P. aeruginosa* has three QS systems, termed *las*, *rhl*, and *pqs* [12]*.* LasI, RhlI, and PqsA are responsible for the biosynthesis of signal molecules N-(3-oxododecanoyl)-L-homoserine lactone (OdDHL), N-butyrylhomoserine lactone (BHL), and Pseudomonas quinolone signal (PQS), respectively [2]. The corresponding receptors for the signal molecules are LasR, RhlR, and PqsR, respectively. Specific autoinducer binding to its receptor can activate genes responsible for pathogenic phenotypes of *P. aeruginosa* [13]*.* Therefore, utilization of anti-QS strategies presents a potential approach to prevent and treat *P. aeruginosa* infections.

In this study, we investigated the antimicrobial and anti-quorum sensing function of hypertonic glucose on *P. aeruginosa* PAO1 and four multidrug-resistant *P. aeruginosa* clinical strains isolated from diabetic foot ulcers, to offer new insights in chronic wound infection treatment.

## Results

### Antimicrobial susceptibility profiles and antimicrobial activity of hypertonic glucose

**Fig. 1:**
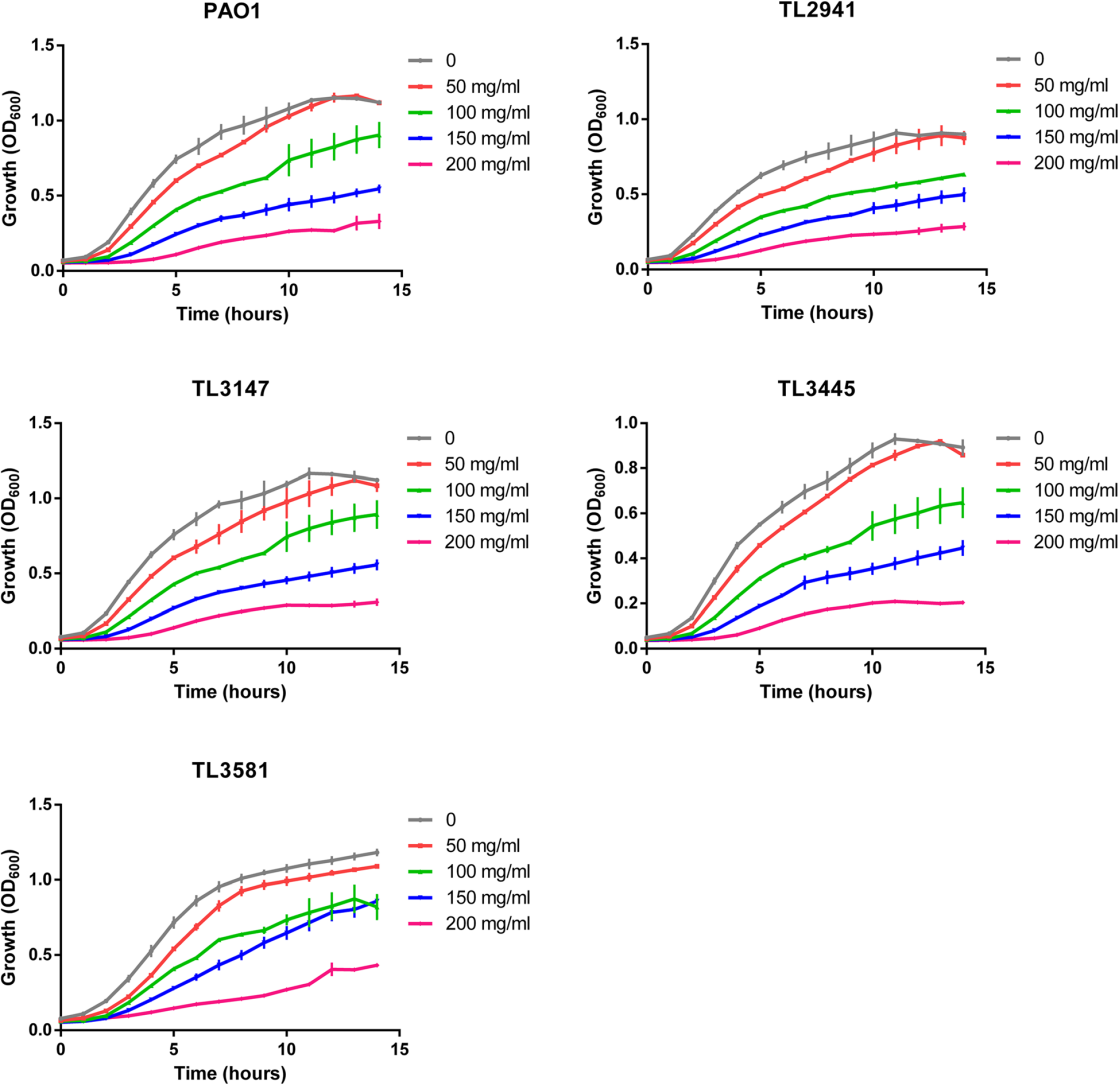
Effects of hypertonic glucose on the growth of *P. aeruginosa* PAO1 and clinical strains*.* Hypertonic glucose showed a concentration-dependent inhibitory effect on the growth of all *P. aeruginosa* strains (*P* < 0.05). Results are expressed as means ± SD (*n* = 3). A ‘no glucose’ group was used as a control

Antibiotic resistance profiles of four multidrug-resistant *P. aeruginosa* strains are displayed in Table 1. All isolates showed resistance to tobramycin, gentamicin, and ciprofloxacin. The broth micro-dilution method was used to determine the MICs of glucose against different strains. As shown in Table 2, glucose inhibited the growth of all the *P. aeruginosa* strains (PAO1, TL2941, TL3147, TL3445 and TL3581) at a concentration of 300 mg/mL. In addition, hypertonic glucose showed a concentration-dependent inhibitory effect on the growth of all *P. aeruginosa* strains at a concentration of at least 50 mg/mL(*P* < 0.05) (Fig. 1).

**Table 1:** Summary of antibiotic resistance profiles of multidrug-resistant *Pseudomonas aeruginosa*

Strains	Antimicrobial resistance										
	AMK	TOB	GEN	CAZ	FEP	ATM	IPM	TZP	SCF	CIP	LVX
TL2941	S	R	R	I	I	R	I	R	I	R	R
TL3147	R	R	R	S	R	R	S	R	I	R	I
TL3445	S	R	R	R	R	N	R	I	S	R	R
TL3581	R	R	R	S	R	N	S	R	I	R	R

*AMK* Amikacin, *TOB* Tobramycin, *GEN* Gentamicin, *CAZ* ceftazidime, *FEP* Cefepime, *ATM* Aztreonam, *IPM* imipenem, *TZP* Piperacillin-tazobactam, *SCF* Cefoperazone-sulbactam, *CIP* ciprofloxacin, *LVX* Levofloxacin, *S* susceptible, *R* resistant, *I* intermediate, *N* Not done

**Table 2:** Minimum inhibitory concentrations of glucose against MDR *Pseudomonas aeruginosa* clinical isolates and PAO1

	Strains	Glucose MIC values (mg/ml)
MDR clinical isolates	TL2941	300
	TL3147	300
	TL3445	300
	TL3581	300
Reference isolates	PAO1	300

### Hypertonic glucose inhibits biofilm formation and swimming motility of *P. aeruginosa*

**Fig. 2:**
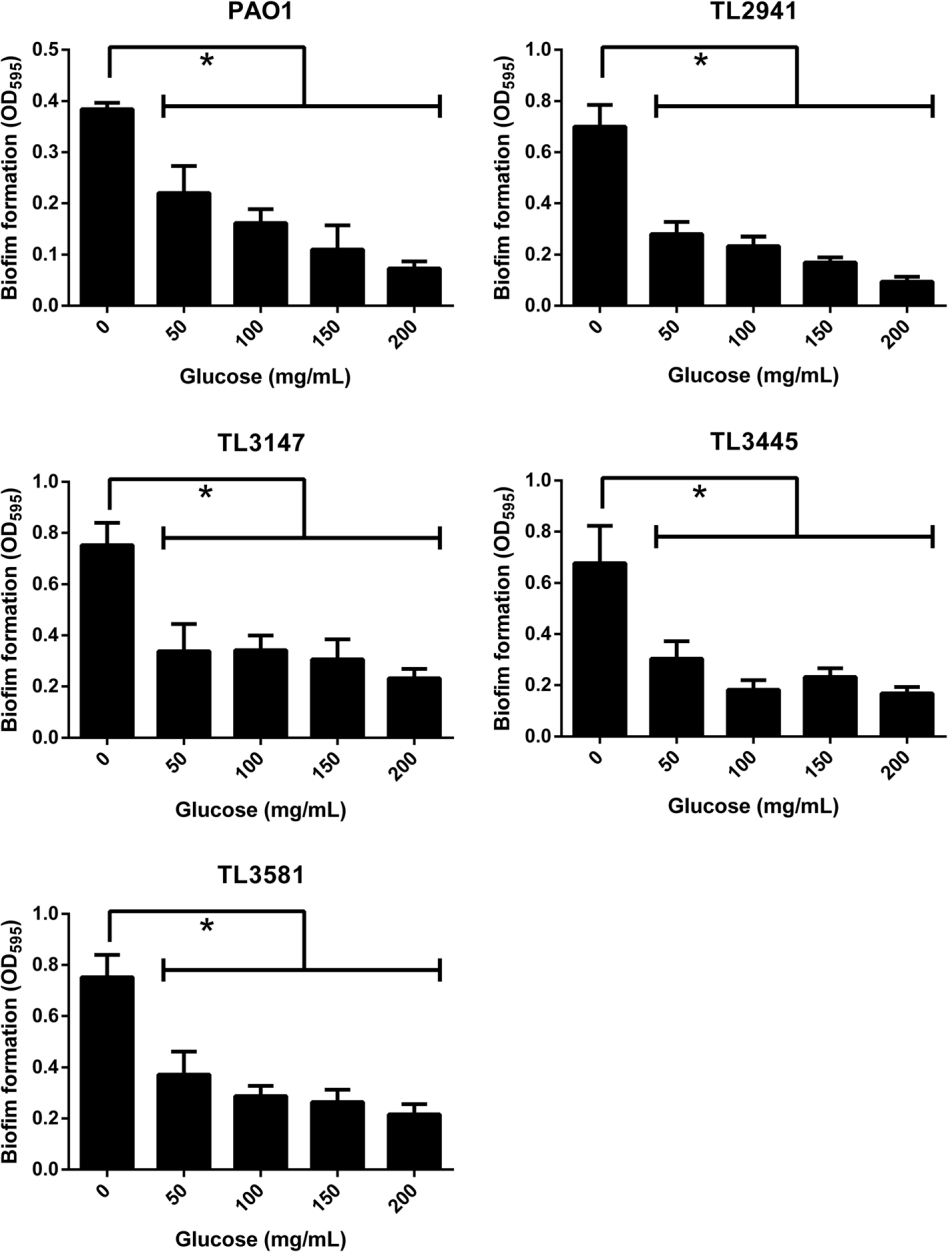
Effects of hypertonic glucose on biofilm formation of *P. aeruginosa*. Hypertonic glucose decreased the biofilm-forming capacity of all tested *P. aeruginosa*. Results are expressed as means ± SD (*n* = 3). * represents *P* < 0.05. A ‘no glucose’ group was used as a control

**Fig. 3:**
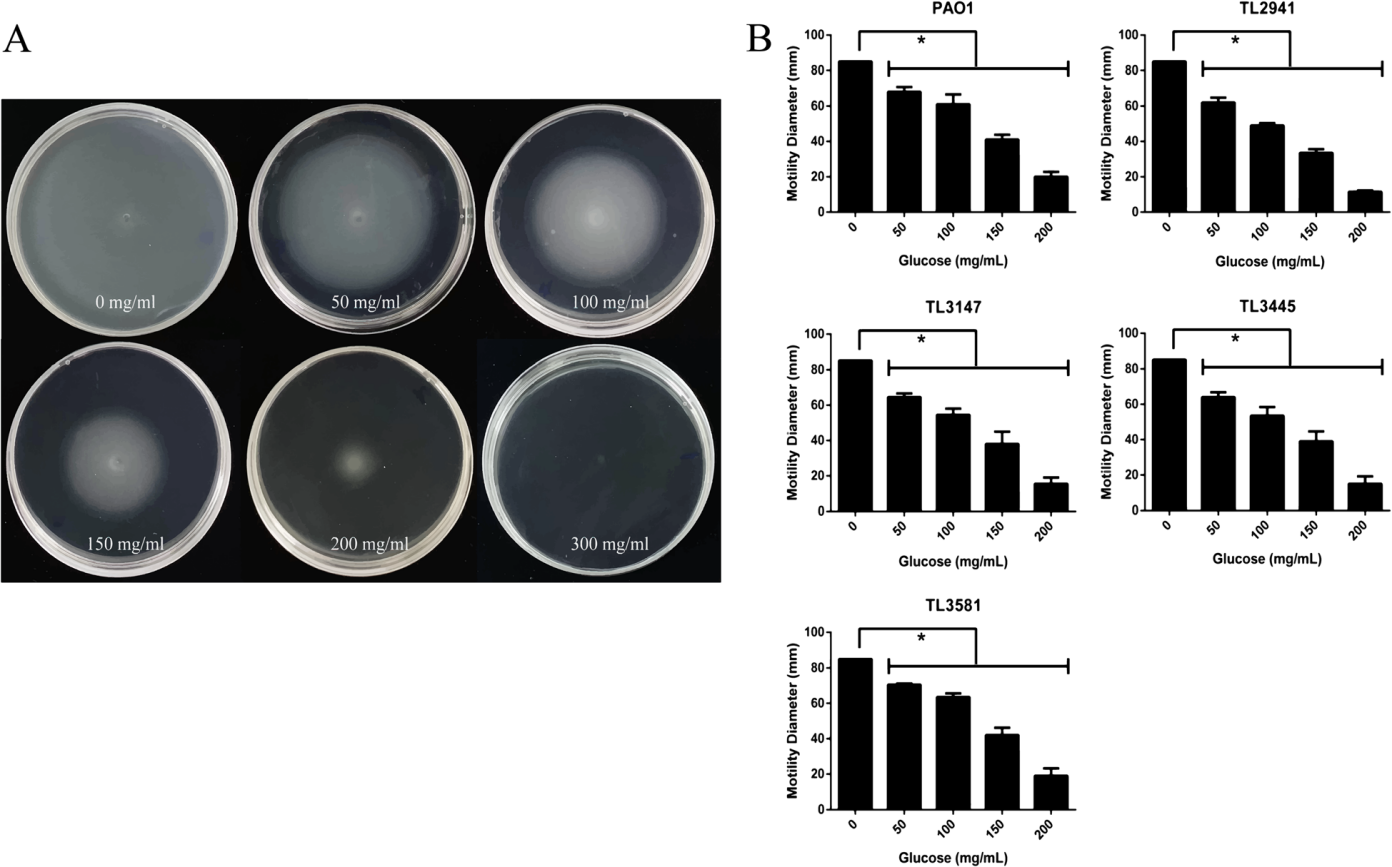
Effects of hypertonic glucose on the swimming motility of *P. aeruginosa* strains (*P* < 0.05) **a**: Swimming agar containing a series of concentrations of hypertonic glucose (0, 50, 100, 150, 200 and 300 mg/mL) inoculated with PAO1. (Image A was spliced with six swimming agar pictures) **b**: Average diameter of the bacterial colony. Results are expressed as means ± SD (*n* = 3). * represents *P* < 0.05. A ‘no glucose’ group was used as a control

The effects of hypertonic glucose on biofilm formation were studied in *P. aeruginosa* using a crystal violet assay. Hypertonic glucose at a concentration of 50 mg/mL caused almost 50% reduction in the biofilm-forming capacity of all tested *P. aeruginosa* isolates (*P* < 0.05) (Fig. 2). Furthermore, hypertonic glucose showed a concentration-dependent inhibitory effect on the swimming motility of all *P. aeruginosa* strains (*P* < 0.05) (Fig. 3). In media containing 300 mg/mL glucose, a circular turbid zone was not observed, demonstrating that motility was completely inhibited at this concentration.

### Hypertonic glucose inhibits virulence factor production

**Fig. 4:**
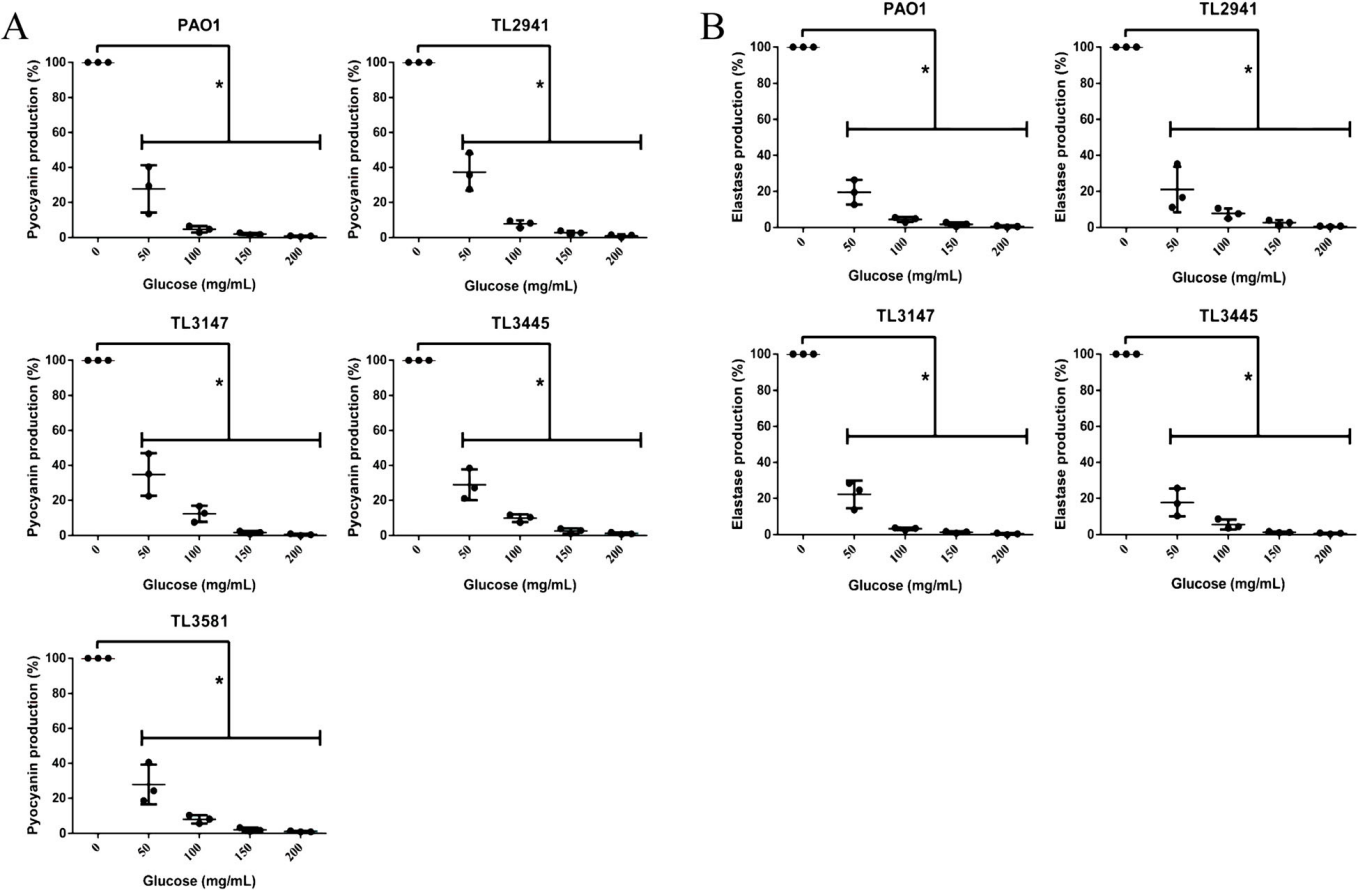
Effects of hypertonic glucose on (**a**) pyocyanin production and (**b**) elastase production of *P. aeruginosa.* Results are expressed as means ± SD (*n* = 3). A ‘no glucose’ group was used as a control. * represents *P* < 0.05. The percentage inhibition rate was not calculated in TL3581 due to limited elastase expression, which could not be detected by the Elastin-Congo red method

The effects of hypertonic glucose on virulence factors such as elastase and pyocyanin were also examined. Hypertonic glucose significantly inhibited the production of elastase and pyocyanin in *P. aeruginosa* PAO1 and strains TL2941, TL3147, and TL3445 at all tested concentrations (Fig. 4). A glucose concentration of 100 mg/mL caused at least 80% reduction in the production of pyocyanin and elastase (*P* < 0.05). In *P. aeruginosa* strain TL3581, the production of pyocyanin was reduced similarly upon hypertonic glucose treatment. However, due to the low levels of elastase produced by TL3581 even in the absence of glucose, which could not be detected by the Elastin-Congo red method, the percentage inhibition rate could not be measured.

### Hypertonic glucose regulates QS-related gene expression

**Fig. 5:**
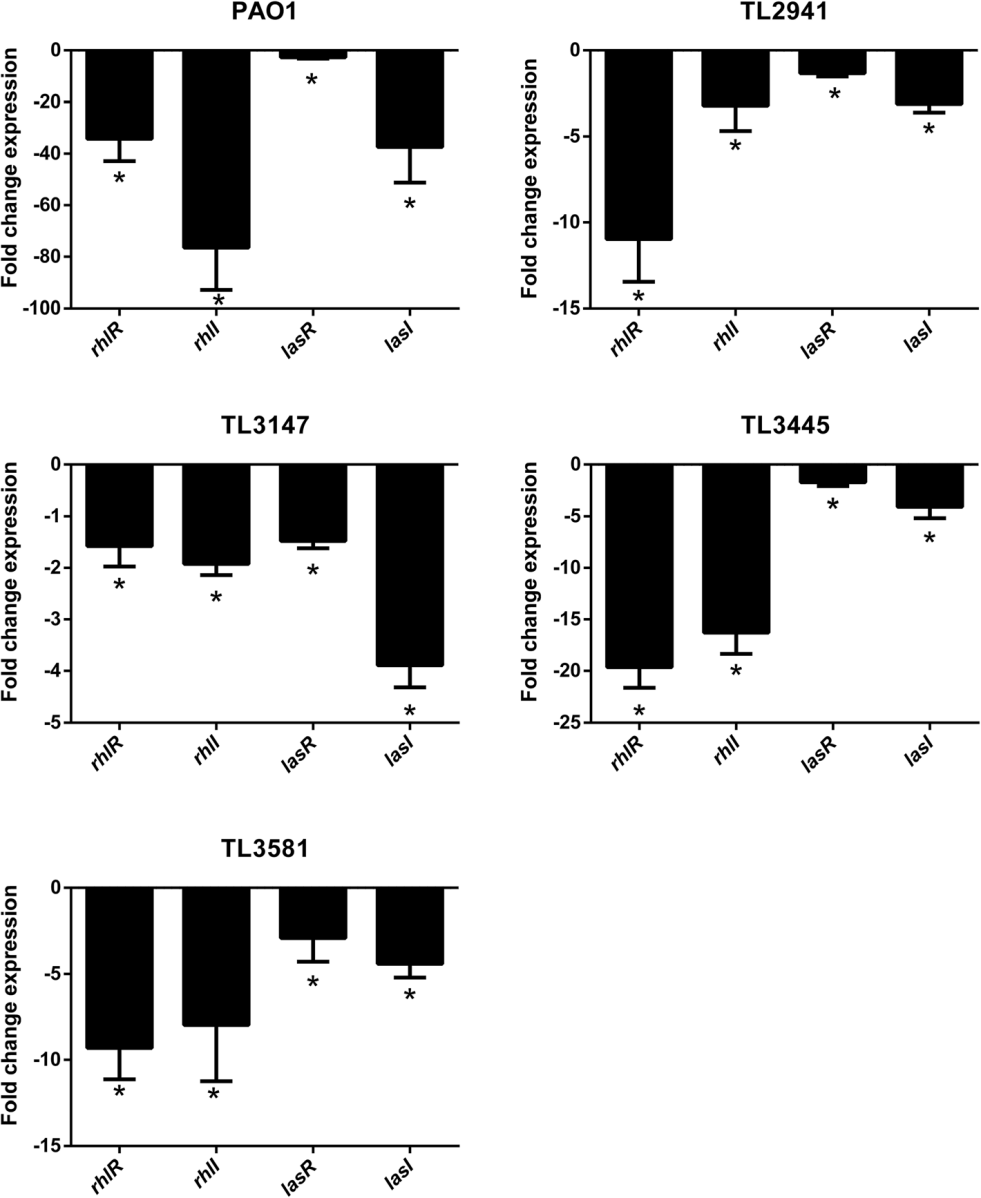
Effects of hypertonic glucose on QS-related gene expression in *P. aeruginosa*. Average relative amounts of tested genes were normalized to the average relative amount of the *rpsL* reference gene. Results are expressed as means ± SD (*n* = 3). A ‘no glucose’ group was used as a control. * represents *P* < 0.05

We next investigated the effect of hypertonic glucose on the expression of QS-related genes, which are commonly associated with the regulation of virulence gene expression and biofilm formation. Therefore, we evaluated the expression of QS-regulated genes in *P. aeruginosa* in response to exposure to hypertonic glucose. The relative expression of genes *lasI*, *lasR*, *rhlI*, and *rhlR* in *P. aeruginosa* PAO1 and multidrug-resistant strains were determined from calculated Ct values. As shown in Fig. 5, QS-related genes expressions showed at least one-fold reduction in all tested *P. aeruginosa* strains under 200 mg/mL hypertonic glucose conditions (*P* < 0.05). Moreover, the relative expressions of genes belonging to the *rhl* regulatory system was lower compared to the *las* regulatory system in *P. aeruginosa* strains except for TL3147.

### Hypertonic glucose reduces mortality in *G. mellonella* larvae infection model

**Fig. 6:**
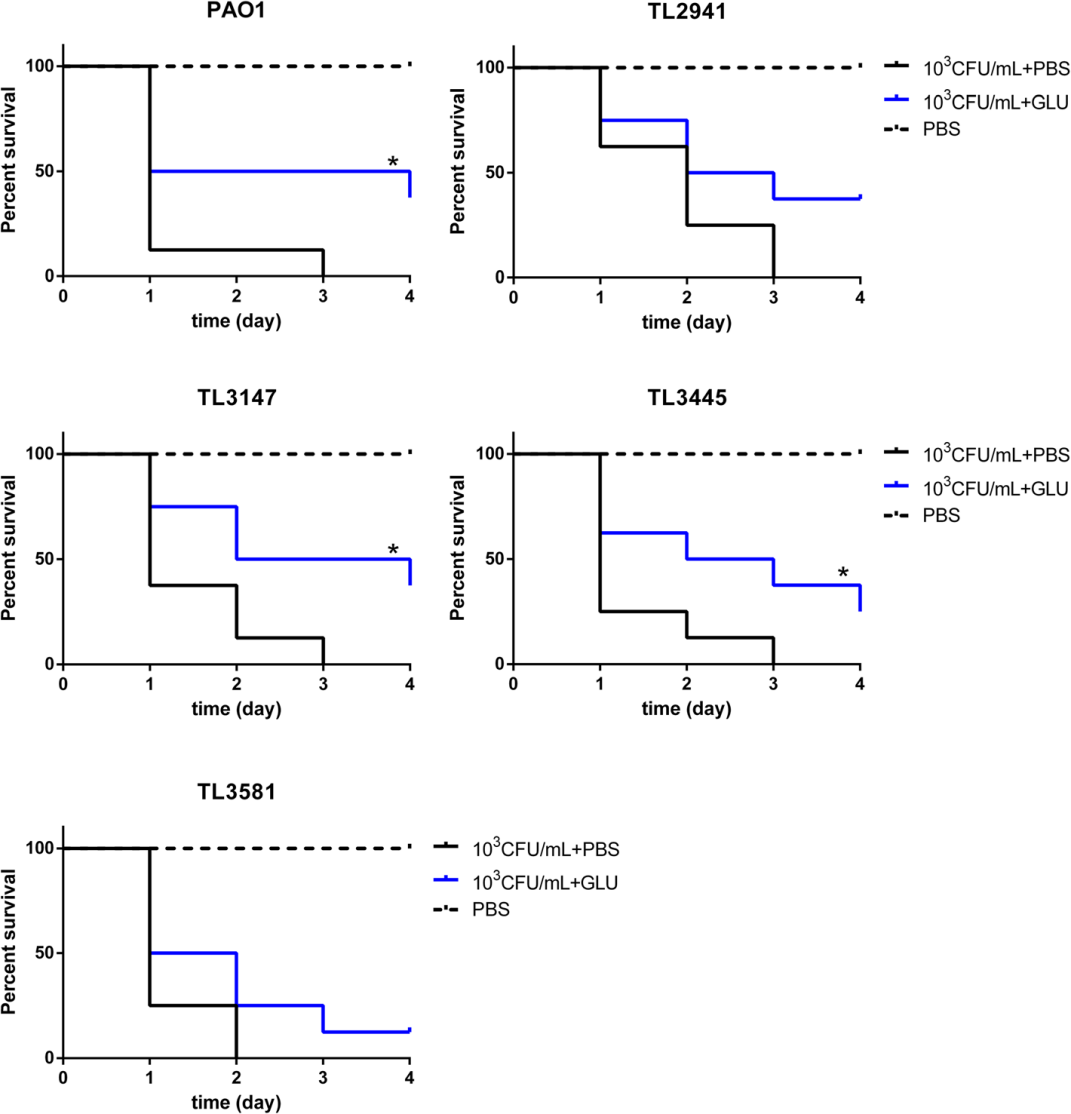
*G. mellonella* killing assays. Effects of hypertonic glucose on the survival rate of larvae. A PBS group was used as a control. A 10 μL of glucose at 200 mg/mL (or a 10 μL of PBS as a control) was administered. Kaplan-Meier analysis and a log-rank test were used to assess the mortality of *G. mellonella.* * indicates a group with significantly enhanced survival compared with the *P. aeruginosa*-infected group treated with PBS (*P <* 0.05)

To investigate the effect of hypertonic glucose on virulence, an in vivo *G. mellonella* infection model was used. All *P. aeruginosa* isolates caused a lethal infection in the larvae (Fig. 6). Following the administration of 200 mg/mLglucose, the survival rates of larvae infected by *P. aeruginosa* isolates (PAO1, TL3147, TL3445) were enhanced (*P <* 0.05). For *P. aeruginosa* strains TL2941 and TL3581, though the survival rates of larvae slightly increased, no significant difference in mortality was displayed after the administration of 200 mg/mL glucose (*P* > 0.05).

## Discussion

*P. aeruginosa* is among the top three major human opportunistic pathogens that cause mortality and morbidity in immunocompromised patients, and is a common cause of chronic wound infections, such as diabetic foot infections [14] and leg ulcers [15]. *P. aeruginosa* has acquired high levels of drug resistance from the widespread use of antibiotics, and new treatment approaches are urgently needed. Hypertonic glucose is a commonly used prolotherapy solution that can reduce healing times; however, it is unclear how hypertonic glucose acts to reduce bacterial virulence associated with wound infections. To date, this is the first study investigating the effects of hypertonic glucose on *P. aeruginosa.*

In this study, we demonstrated that hypertonic glucose had an inhibitory effect on the growth of *P. aeruginosa* PAO1 and four clinical strains at a concentration of more than 50 mg/mL. We further explored the actions of glucose activity against *P. aeruginosa* at sub-MICs. The pathogenesis of *P. aeruginosa* relies largely on motility and the production of virulence factors such as pyocyanin and elastase, which are controlled by QS systems [16]. We observed that hypertonic glucose significantly inhibited swimming motility in a concentration-dependent manner and reduced the production of pyocyanin and elastase by over 80% when used at a concentration of 100 mg/mL, which demonstrated a potential role of hypertonic glucose to reduce *P. aeruginosa* virulence. It is known that *P. aeruginosa* frequently forms biofilms on medical surfaces and is associated with chronic wound infections. Such biofilms are highly refractory to removal and resistant to antimicrobials, and their presence is generally correlated with poor treatment outcomes [17, 18]. This study revealed that biofilm formation of all tested *P. aeruginosa* strains was attenuated in the presence of hypertonic glucose.

Targeting QS systems represents an attractive option for infection control, which could minimize selection pressures and the risk of drug resistance. In this work, we investigated whether the expressions of major QS-regulated genes *lasI*, *lasR*, *rhlI*, and *rhlR* could be influenced by hypertonic glucose treatment to attenuate *P. aeruginosa* virulence factors. We showed that hypertonic glucose treatment significantly reduced the expression of *lasI*, *lasR*, *rhlI*, and *rhlR* genes (*P* < 0.05). In the *las* and *rhl* systems, the virulence factors of *P. aeruginosa* are primarily encoded by QS-regulated genes *lasI*, *lasR*, *rhlI*, and *rhlR* [16]*.* The *lasI* and *rhlI* genes are required for the synthesis of QS signals, which are capable of binding to the transcriptional regulator LasR and RhlR, respectively. It is known that LasR and RhlR interacting with the corresponding signals can trigger the production of pyocyanin and elastase in *P. aeruginosa* [19]. In this study, the significantly decreased expression of QS-regulated genes correlated with a decrease in virulence factor production. A qRT-PCR experiment showed that the relative expression of genes belonging to the *rhl* regulatory system was reduced more than the *las* regulatory system in *P. aeruginosa* strains, except TL3147 (Fig. 5). This was an interesting finding, which indicates that the *rhl* regulatory system might be more sensitive to hypertonic glucose treatment. Further studies are required to identify the mechanisms involved.

We used a *G. mellonella* infection model to explore the anti-virulence ability of hypertonic glucose in vivo. The results showed hypertonic glucose reduced the mortality in *G. mellonella* larvae infection model for a majority of strains (PAO1, TL3147, and TL3445). However, no significant change in mortality of *G. mellonella* larvae was observed with the administration of hypertonic glucose for other two isolates (TL2941 and TL3581). It is possible that once the in vivo levels of hypertonic glucose drop below a certain threshold, the growth inhibition and virulence factor expression of *P. aeruginosa* is rapidly alleviated, and its pathogenicity restored. The reason for the differences between strains will be further explored.

It is known that QS systems play an important role in wound infections [20]. The exploitation of QS systems to search for virulence factor inhibitors has offered an alternative opportunity to control bacterial infections, given the fact that traditional antimicrobial treatments can easily become ineffective due to bacterial resistance. QS inhibitors have been shown to greatly impair bacterial infections, especially in the control of biofilm-related diseases [21, 22]. We showed here that hypertonic glucose could inhibit growth, attenuate virulence factors, and regulate quorum sensing of multidrug-resistant *P. aeruginosa*. Based on this notion, further strategies involving hypertonic glucose should be considered to enhance the therapeutic effect of treatments to combat bacterial infections.

## Conclusions

In conclusion, to the best of our knowledge, this is the first study to report the inhibitory effects of hypertonic glucose on *P. aeruginosa*. Hypertonic glucose displayed the ability to inhibit growth, biofilm formation, and swimming motility as well as attenuate virulence factors of *P. aeruginosa*. A reduced expression of genes involved in quorum sensing correlated with attenuated virulence factors. Future studies that investigate the activity of hypertonic glucose should be considered in wound infection management.

## Methods

### Bacterial strains

*P. aeruginosa* strain PAO1, and four multidrug-resistant *P. aeruginosa* strains (TL2941, TL3147, TL3445 and TL3581) isolated from different diabetic foot ulcers from patients at the First Affiliated Hospital of Wenzhou Medical University in China were used in this study. Identification and antimicrobial susceptibility testing were conducted on clinical isolates using the VITEK MS and VITEK2 systems, respectively. All strains were cultured in LB medium (1% tryptone, 1.0% NaCl, 0.5% yeast extract) at 37 °C.

### Minimum inhibitory concentration assay

The minimum inhibitory concentration (MIC) values of glucose (D-(+)-glucose; Sigma, St. Louis, USA) were determined using previously published methods, with some modifications [23]. Briefly, bacterial suspensions (10^8^ CFU/mL) of *P. aeruginosa* PAO1 and multidrug-resistant *P. aeruginosa* strains were added to LB broth (1%, v/v) that contained glucose at concentrations of (0, 50, 100, 150, 200, 250, 300 and 350 mg/mL) in 96-well microtiter plates, and then incubated at 37 °C for 18 h. The MIC was the lowest glucose concentration that caused complete inhibition of cell growth. All further experiments in this study used sub-minimum inhibitory concentrations (sub-MICs) of glucose.

### Growth curve assay

The effects of glucose on the growth rate of *P. aeruginosa* strains were determined according to a method described previously, with some modifications [24]. Briefly, overnight cultures were diluted 1:100 into fresh LB broth and incubated at 37 °C for 4 h before adjusting the OD_600_ values to 0.1. A 100 μL aliquot of the subcultures were added to 96-well microtiter plates containing 100 μL LB with different concentrations of glucose (0, 100, 200, 300 and 400 mg/mL). LB medium and LB with glucose were used as negative controls. The microtiter plates were incubated at 37 °C, with shaking at 180 rpm. The absorbance of each sample at OD_600_ was measured every 1 h.

### Biofilm formation assay

The crystal violet method was used to measure the biofilm-forming capacity of *P. aeruginosa* strains. Strains were initially grown in LB broth for 17 h, which were then diluted 1:100 in fresh LB broth supplemented with glucose (0, 50, 100, 150 and 200 mg/mL). A 100 μL aliquot of each sample was then transferred to a 96-well microtiter plate and incubated at 37 °C for 24 h. Wells containing media alone were used as references. Planktonic cells were removed, and the wells were washed twice with sterile water. Wells were then stained with 150 μL 0.1% crystal violet for 10 min and rinsed twice with sterile water. Stained biofilms were solubilized with 95% ethanol and quantified by measuring the OD_595_ using a microplate reader.

### Swimming motility assay

The swimming assay was conducted as described elsewhere, with some modifications [23]. Briefly, glucose solutions at different concentrations (0, 50, 100, 150, 200 and 300 mg/mL) were added to molten swimming agar, which consisted of 0.1% tryptone, 0.05% yeast extract, 0.5% NaCl and 0.3% bacteriological agar (pH 7.2). A 2 μL aliquot of *P. aeruginosa* culture (≈10^7^ CFU) was inoculated in the center of the agar and then incubated at 37 °C for 24 h.

### Pyocyanin assay

Pyocyanin production was examined as described previously with modifications [25]. Bacterial cultures containing different concentrations of glucose (0, 50, 100, 150 and 200 mg/mL) were grown for 17 h, and 1 mL samples were then centrifuged at 13,000 rpm for 5 min. The supernatant was collected and extracted with 600 μL chloroform, and then mixed by vortex (2 × 10 s). After centrifugation at 13,000 rpm for 5 min, the chloroform phase was transferred to a sterile tube and mixed with 0.5 mL of 0.2 M HCl followed by gentle shaking to transfer the pyocyanin to the aqueous phase. Pyocyanin was determined by measuring the absorbance of the aqueous phase at OD_510_.

### Elastase activity assay

The elastolytic activity of the cell-free culture supernatant of *P. aeruginosa* was determined using Elastin-Congo red (ECR; Sigma) as the substrate [26]. 100 μL supernatants of *P. aeruginosa* incubated in different concentrations of glucose (0, 50, 100, 150 and 200 mg/mL) for 17 h were added to 900 μL of ECR buffer (100 mM Tris, 1 mM CaCl_2_, pH 7.5) containing 10 mg of ECR and incubated at 37 °C for 3 h. The reaction was terminated by adding 1 mL of 0.7 M sodium phosphate buffer (pH 6.0) and the tubes were placed in a cold water-bath. The insoluble ECR was removed by centrifugation at 10,000 rpm for 10 min, and the absorbance was measured at OD_495_.

### *Galleria mellonella* larvae infection model

*G. mellonella* killing assays were performed as described previously, with some modifications [27]. Eight larvae weighing between 200 and 250 mg were randomly selected for each *P. aeruginosa* strain. Overnight bacterial cultures were diluted 1:100 in fresh LB broth and incubated at 37 °C for 4 h. Cell suspensions were washed three times with phosphate-buffered saline (PBS) and resuspended in PBS to 10^3^ CFU/mL. A 10 μL of bacterial suspension (10^3^ CFU/mL) was injected into the last left proleg using a 25 μL Hamilton precision syringe and a 10 μL of glucose at 200 mg/mL (or a 10 μL of PBS as a control) was injected into the last right-side proleg at 2 h post infection. Larvae injected with 10 μl PBS were used as controls. *G. mellonella* were incubated at 37 °C in the dark and observed after 24 h, 48 h, 72 h and 96 h. Larvae were considered dead when they repeatedly failed to respond to physical stimuli. The primary outcome for the insect model was rapidity and extent of mortality of *G. mellonella*, as assessed with Kaplan-Meier analysis and a log-rank test.

### Quantitative reverse transcription PCR

The effects of hypertonic glucose on the expression levels of *P. aeruginosa* major QS circuit genes (*lasI, lasR, rhlI,* and *rhlR*) were evaluated using quantitative reverse transcription PCR (qRT-PCR). RNA was extracted from *P. aeruginosa* isolates that were grown in fresh LB medium with or without 200 mg/mL glucose at 37 °C for 17 h. Total RNA was extracted from planktonic bacteria using an RNeasy Mini Kit (Qiagen, Valencia, CA, USA) according to the manufacturer’s instructions. The extracted RNA samples were stored at − 80 °C. Purified RNA was reverse transcribed into cDNA using a cDNA Synthesis Kit (TaKaRa, Tokyo, Japan) according to the manufacturer’s instructions. Gene expression levels were measured with qRT-PCR using a 7500 RT-PGE system (TOYOBO, Osaka, Japan) and a SYBR Green qRT-PCR Kit (TOYOBO) with specific primers listed in Additional Table S1. The *rpsL* gene was used as an internal control to normalize the data. Gene expression levels were calculated using the 2^−△△Ct^ method.

### Statistical analysis

All experiments were conducted independently with at least three replicates on different days, and results were expressed as mean ± standard deviation. The total area under the curve was calculated for the growth rate analysis. The results of the pyocyanin and elastase assays are presented as production percentage of the untreated (0 mg/ml) samples. A T-test was used to evaluate the significance of differences between two groups. One-way analysis of variance (ANOVA) was performed to analyze the significance among more than two groups. Statistical significance was determined at *P* < 0.05. Statistical analyses were performed using SPSS version 17.0 statistical software.

## Supplementary information

**Additional file 1:.** Additional Table S1

## Data Availability

The datasets used and analyzed during the study are available from the corresponding author on reasonable request.

## Abbreviations

qRT-PCR: Quantitative Reverse Transcription Polymerase Chain Reaction
QS: Quorum sensing
OdDHL: N-(3-oxododecanoyl)-L-homoserine lactone
BHL: N-butyrylhomoserine lactone
PQS: Pseudomonas quinolone signal
