# Supplementary material for: Hypertonic glucose inhibits growth and attenuates virulence factors of multidrug-resistant Pseudomonas aeruginosa
Source: BMC Microbiol. 2020 Jul 9;20:203. doi: 10.1186/s12866-020-01889-2 (PMC7346426; doi:10.1186/s12866-020-01889-2)
Supplement: Supplementary file 1 — Additional file 1:. Additional Table S1 [file 12866_2020_1889_MOESM1_ESM.docx]

| **Gene Name** | **Type** | **Primer sequence** | **Annealing temp (°C)** | **Amplicon size (bp)** |
| --- | --- | --- | --- | --- |
| *lasI* | F | 5‘- GGCTGGGACGTTAGTGTCAT-3’ | 58 | 104 |
|  | R | 5‘-AAAACCTGGGCTTCAGGAGT-3’ |  |  |
| *lasR* | F | 5‘-ACGCTCAAGTGGAAAATTGG-3’ | 58 | 111 |
|  | R | 5‘-TCGTAGTCCTGGCTGTCCTT-3’ |  |  |
| *rhlI* | F | 5‘-AAGGACGTCTTCGCCTACCT-3’ | 58 | 130 |
|  | R | 5‘- GCAGGCTGGACCAGAATATC-3’ |  |  |
| *rhlR* | F | 5‘-CATCCGATGCTGATGTCCAACC-3’ | 60 | 101 |
|  | R | 5‘-ATGATGGCGATTTCCCCGGAAC-3’ |  |  |
| *rpsL* | F | 5‘-GCAACTATCAACCAGCTGGTG-3’ | 58 | 231 |
|  | R | 5‘-GCTGTGCTCTTGCAGGTTGTG-3’ |  |  |

**Additional Table S1. Primers used in qRT-PCR for quorum sensing circuit genes *lasI*, *lasR*, *rhlI,* and *rhlR*, and reference gene, *rpsL*.**
